# Impairment of 7F2 osteoblast function by simulated partial gravity in a Random Positioning Machine

**DOI:** 10.1038/s41526-022-00202-x

**Published:** 2022-06-07

**Authors:** Justin Braveboy-Wagner, Peter I. Lelkes

**Affiliations:** Department of Bioengineering, College of Engineering, Temple University, Philadelphia, PA USA

**Keywords:** Biomaterials - cells, Prognostic markers, Cell biology

## Abstract

The multifaceted adverse effects of reduced gravity pose a significant challenge to human spaceflight. Previous studies have shown that bone formation by osteoblasts decreases under microgravity conditions, both real and simulated. However, the effects of partial gravity on osteoblasts’ function are less well understood. Utilizing the software-driven newer version of the Random Positioning Machine (RPM^SW^), we simulated levels of partial gravity relevant to future manned space missions: Mars (0.38 G), Moon (0.16 G), and microgravity (Micro, ~10^−3^ G). Short-term (6 days) culture yielded a dose-dependent reduction in proliferation and the enzymatic activity of alkaline phosphatase (ALP), while long-term studies (21 days) showed a distinct dose-dependent inhibition of mineralization. By contrast, expression levels of key osteogenic genes (Alkaline phosphatase, Runt-related Transcription Factor 2, Sparc/osteonectin) exhibited a threshold behavior: gene expression was significantly inhibited when the cells were exposed to Mars-simulating partial gravity, and this was not reduced further when the cells were cultured under simulated Moon or microgravity conditions. Our data suggest that impairment of cell function with decreasing simulated gravity levels is graded and that the threshold profile observed for reduced gene expression is distinct from the dose dependence observed for cell proliferation, ALP activity, and mineral deposition. Our study is of relevance, given the dearth of research into the effects of Lunar and Martian gravity for forthcoming space exploration.

## Introduction

Loss of structural skeletal mineral is a health complication facing any human presence in space or, to a yet unknown degree, on other terrestrial bodies with reduced gravity, like the Moon (~0.16 G) or Mars (~0.38 G). The detrimental effects of microgravity on the musculoskeletal system have been known, albeit not fully understood, since the onset of manned spaceflight. The effect was confirmed as astronauts and cosmonauts began to spend increasingly more time in space^1^. In studies conducted on the International Space Station (ISS), significant trabecular volumetric bone mineral density (vBMD) losses in the spine and hip were noted with femoral vBMD showing an average loss of 2.7%/month and losses at the trabecular hip of 2.3%/month^2^. If not mitigated by proper countermeasures, loss of bone mass poses an increased risk of fracture in the workplace (space) and negatively impacts quality of life on return to Earth.

The primary cause of bone loss in microgravity (and presumably also under the partial-gravity conditions found on Mars or Moon) is believed to be the inhibition of osteoblast and osteocyte activity, resulting in decreased bone mineralization^3^, concomitant with elevated osteoclast resorption. The upset equilibrium results in a substantial loss of bone mass over time, as mentioned above. The effects of microgravity on osteoblasts have been previously explored: in experiments carried out in orbital microgravity (at ~10^−4^–10^−6^ G)^4^ osteoblast proliferation was inhibited, osteogenic differentiation was delayed, and the expression of genes controlling bone differentiation was reduced^5–7^, while the bone resorption by osteoclasts increased aggressively^8,9^. The result is an overall loss of bone mineral density, wherein load-bearing bones are prone to atrophy^10^, although one study reports a small increase in skull bone mineral density^11^. These effects have been replicated when osteoblasts and preosteoblasts were cultured on earth in microgravity analogs (rotating wall vessel (RWV) bioreactors and clinostats) that recapitulate certain aspects of microgravity. These experiments demonstrated that simulated microgravity (Micro) conditions (equivalent to ~10^−3^ G^12,13^), suppress osteoblast differentiation^12,14^ and alter osteoclast function and survival^15^, similar to the effects observed in orbital microgravity, making these venues cost-effective substitutes for gravity modeling and experimentation.

While alternatives like parabolic flights exist for short-term studies, the experimental gold standards for simulating microgravity *on Earth* are rotating clinostats^16^ or microgravity-simulating bioreactors^12,17^. There is ample evidence in the literature suggesting that the results of ground-based modeled microgravity studies mimic real space conditions^18–20^. In comparative studies, similar degrees of inhibition of proliferation and differentiation have been observed both in osteoblast-like cells cultured in true-microgravity in space as well as in experiments carried out in simulated microgravity using clinostats^21^. The Random Positioning Machine (RPM), a 3D clinostat, randomizes orientation with respect to Earth’s gravitation field so that a sample’s gravity vector averages out to close to zero, providing a state of simulated microgravity^22^. Using outer and inner frames, fixed to two separate axes, the RPM can rotate independently in three dimensions and simulate partial gravities beyond microgravity, such as Moon and Mars. This is an advantage the RPM has over the two-dimensional rotation of the rotating wall vessel (RWV) or 2D clinostat. In vitro studies carried out in the RPM have yielded results similar to those performed in actual (orbital) microgravity in bone-marrow-derived mesenchymal stem cells (MSCs)^23^ and 2T3 preosteoblasts^24^. An alternative approach to the operation of an RPM (RPM^SW^) is to control the range of clinostat motion through the use of specific path files that move the arms of the device through pre-determined paths. These paths are not random, rather they are set for every iteration of the experiment, allowing for the simulation of distinct partial gravities: in our case simulating the reduced gravity levels encountered for Mars, Moon, and Microgravity^25–27^.

For the sake of scientific rigor and within the context of this paper, the term Simulated Partial Gravity (SPG) or Simulated Microgravity (SMG) refers specifically to the respective reduction in the net gravitational vector as generated by the motion-averaged movement of the 3D clinostat or RPM. The use of the terms “Mars” and “Moon” does not indicate that we actually recreated the respective extraterrestrial partial-gravity conditions. Rather, operating the RPM in the specific path files generates earth-based analog environments that simulate many of the environmental conditions expected at 0.38 G (Mars) and 0.16 G (Moon).

In this study, we cultured murine preosteoblasts (7F2 cells) in the RPM under conditions that simulate microgravity (10^−3^ G), and the partial gravities on Moon (0.16 G), and Mars (0.38 G). Specifically, we mostly focused on the effects of altered simulated gravity conditions on several distinct stages of initial osteoblastic cell functions, such as proliferation and osteogenic differentiation^28^. In addition, we also evaluated the effects of these simulated partial-gravity conditions on later-stage matrix mineralization. We hypothesized that the inhibition of these stage-specific osteoblast functions would depend on the simulated partial-gravity levels. In testing this hypothesis, we observed that the inhibition of two of these osteoblast functions, proliferation, and maturation (ALP-enzymatic activity and mineral deposition) was dose-dependent. By contrast, we found a distinct threshold profile for the inhibition of osteogenic marker gene expression.

## Materials and methods

### Materials

Alpha-minimum essential medium (a-MEM) and Fetal Bovine Serum (FBS) were purchased from Gibco Life Technologies (Carlsbad, CA, USA). L-ascorbic acid (AA), β–glycerophosphate (β-GP), para-Nitrophenylphosphate (pNPP), Alizarin Red, and Tri Reagent® for processing tissues were purchased from Sigma-Aldrich (St. Louis, MO, USA). Quant-iT™ PicoGreen™ dsDNA Assay Kit was purchased from Invitrogen Molecular Probes (Eugene, OR, USA) via Thermo Fisher Scientific. TaqMan Fast Universal PCR Master Mix (2X) and Taqman primers were purchased through Applied Biosystems (Foster City, CA, USA). RNeasy Protect Mini Kits were purchased from Qiagen (Hilden, GER).

### Cell culture techniques

7F2 murine preosteoblasts (American Type Culture Collection, Manassas, VA, USA, CRL-12557) were cultured in α-MEM media supplemented with 10 mM HEPES, 10% FBS, 1% streptomycin and penicillin, and maintained in a humidified, 37 °C, 5% CO_2_/air incubator (maintenance medium). For osteogenic induction, the cells were cultured in osteogenic media containing the above complete alpha-MEM culture medium supplemented with 10 mM β-glycerophosphate and 10 µg/ml ascorbic acid (differentiation medium). The media was changed every 3 days. In addition to frequent equilibrated media changes, oxygenation was maintained by using modified culture flasks utilizing silicon caps in place of ventilated caps, and shear stress was minimized by having the flask completely filled with culture medium and devoid of air bubbles, which are notoriously detrimental to maintaining the slow-shear, simulated microgravity conditions^29^, thus maintaining the concept of near-solid body (“zero headspace”). Cells were cultured as 2D monolayers in T-12.5 Falcon™ Tissue Culture Treated Flasks (Fisher Scientific, Waltham, MA, USA) retrofitted with Fischer-brand Silicone Recessed Septum Stoppers (internal diameter: 14.5–15.5 mm) for enhanced gas exchange. The accessories used are shown in Supplementary Fig. 1, including the 3D printed mounting cage for loading and unloading flasks and the perfusion system used to eliminate bubbles and change media.

### Random Positioning Machine

To simulate reduced gravity, all experiments were carried out in the second generation of the software-driven Random Positioning Machine (RPM^SW^ 2.0) (originally DutchSpace Airbus, Leiden, Netherlands, now Yuri GmbH, Meckenbeuren, Germany). The Mode of operation of this RPM differs from that of the earlier generation, which was developed for generating completely random paths, thus creating a simulated microgravity condition, and used for years in research^18–20^. The “mode of operation” of the RPM^SW^ for simulating micro and partial gravity (see Supplementary Fig. 3) was described by Benavides Damm et al.^30^ and was previously used to test the effects of modeled Mars gravity in plants^25,31^. To simulate partial gravity the RPM utilizes pre-determined path files, developed, and validated by the manufacturer (Dutch Airbus), in which the software directs the motion of the RPM arms in a non-random fashion. These paths will have a degree of preference along the Earth gravity vector, and the result is a net positive gravity that is greater than net-zero but <1 G normal. The motion of the RPM in random mode over time can be visualized as a sphere while the motion over time of a path file can be visualized as a prolate spheroid. In the center of this spheroid, a sample is not weightless, as with a net-zero sphere, but instead experiences partial gravity, where the larger the eccentricity of the spheroid the higher the level of gravity. Additional validation of the simulation of partial gravity via vector averaging was achieved through maintenance software of the RPM, which converts feedback from the frames into a real-time vector average (Supplementary Fig. 4).

### Alkaline phosphatase (ALP) activity assay

Alkaline phosphatase (ALP) enzymatic activity was used as a marker for osteoblastic differentiation and quantitated spectrophotometrically. Following a room-temperature PBS wash, cell monolayers were scraped in 250 µl PBS and transferred into 1 ml microcentrifuge tubes. The cells were then lysed with 250 µl 0.2% Triton in PBS, to a final concentration of 0.1% (v/v) Triton X-100 (500 µl), followed by one freeze-thawing cycle (−80 °C/RT). After thawing and centrifugation (2000 × g, 1 min), the supernatants were used to determine ALP activity according to the protocol of Lin et al.^32^ with some modifications: the buffer used was 10 mM MgCl_2_, 0.5 M AMP (2-Amino-2-Methyl-1-Propanol), supplemented with 9 mM of the ALP substrate, p-nitrophenylphosphate (pNPP). The lysate was diluted 10×. Color development was read in situ in an Infinite 200 PRO multimode plate reader (Tecan Group Ltd., Switzerland) every 2 min for 14 min at 405 nm. Readings were converted to concentration (nM) with a standard curve based on 4-Nitrophenol. ALP results are presented as enzyme activity over time (the rate of p-nitrophenol production from the p-nitrophenylphosphate substrate) and normalized to cell number as calculated from DNA content, using PICO green (as below). The normalized results are expressed as the amount of substrate converted (ng) over time per number of cells (ng/min/10k cells).

### PICO-green assay for cell proliferation

PicoGreen dsDNA Quantitation Reagent (Invitrogen, Eugene OR, USA) was supplied as a 1-ml concentrated dye solution in anhydrous dimethylsulfoxide (DMSO) and used following the manufacturer’s protocol, with precedent in osteoblast studies^33^. 100 µl of the 0.1% Triton monolayer lysate supernatant (see above) was removed, diluted 400×, and added to a 96-well plate. 100 µl of the combined PicoGreen Reagent (1:200 PicoGreen diluted in the TE buffer supplied with the kit) was added to each sample. After mixing and incubation for 5 min at room temperature, protected from light, fluorescence was measured on an Infinite 200 PRO multimode plate reader (Tecan Group Ltd., Switzerland) at 485 nm excitation, 535 nm emission. Standard curves were constructed from known cell numbers (counted in triplicate).

### Alizarin Red and dissolved TECO assay for mineralization

Mineralization of the cultures was assessed qualitatively by Alizarin Red staining, essentially as previously described^34^. In brief, following washing with PBS and fixation with 10% neutral buffered formalin for 15 min, the cultures were stained using 0.5% Alizarin Red S (pH 4.2) Digital images of the stained calcified nodules were evaluated using ImageJ software (National Institutes of Health, NIH). Mineralization was also quantified using a commercially available, colorimetric calcium quantification kit (Teco Diagnostics, Anaheim CA), following destructive decalcification of the cultures in 0.6 N HCl and analyzing the supernatant according to the manufacturer’s instructions. Separate standard curves were established for each experiment.

### RNA extraction and real-time PCR for osteogenic marker gene expression (ALPL, RUN, ON)

The expression of select osteogenic marker genes was determined by quantitative PCR (qPCR) essentially as previously described, with some minor modifications^34^. In brief, total RNA was extracted from 7F2 cells using a modified version of a hybrid Tri Reagent (Sigma-Aldrich, USA)/RNEasy® protocol. RNA was quantified using a NanoDrop Spectrophotometer (Thermo Scientific, Waltham, MA), and concentrations were brought to a uniform level using additional RNase-free water. RNA was reverse transcribed to cDNA using the high-capacity cDNA reverse transcription kit (Applied Biosystems, Foster City, CA) according to the manufacturer’s instructions. The cDNA was amplified in TaqMan Fast universal PCR master mix with TaqMan assay primers and probes according to the manufacturer’s instruction. Genes of interest were: ALPL (Mm00475834_m1), RUNx2 (Mm00501584_m1) and Sparc/osteonectin/BM40 (Mm00486332_m1). Quantitative PCR (qPCR) was performed in a RealPlex Real-Time PCR System (Eppendorf, Enfield, CT) with fast thermal cycling as described by Taqman (Applied Biosystems). The level of expression of each gene was normalized to the level of expression of a common standard housekeeping gene, glyceraldehyde 3-phosphate dehydrogenase (GAPDH) to determine the fold change in up/downregulation of the genes of interest using the comparative CT method (2-ΔΔCT)^35^.

### Statistical analysis

Throughout the text, unless otherwise specified, statistical differences between the samples were assessed by ANOVA and post hoc analysis using Tukey’s HSD (honestly significant difference). Mean absolute error (MAE) was used to determine the difference between modeled projections. Results were plotted using Excel or JMP Pro. Data are presented as means ± standard deviation. *P* < 0.05 was considered significant and noted as “*”, *P* < 0.01 and *P* < 0.001 were noted as “**” and “***”, respectively.

### Reporting summary

Further information on research design is available in the Nature Research Reporting Summary linked to this article.

## Results

### Inhibition of 7F2 cell proliferation in simulated micro and partial gravity

**Fig. 1:**
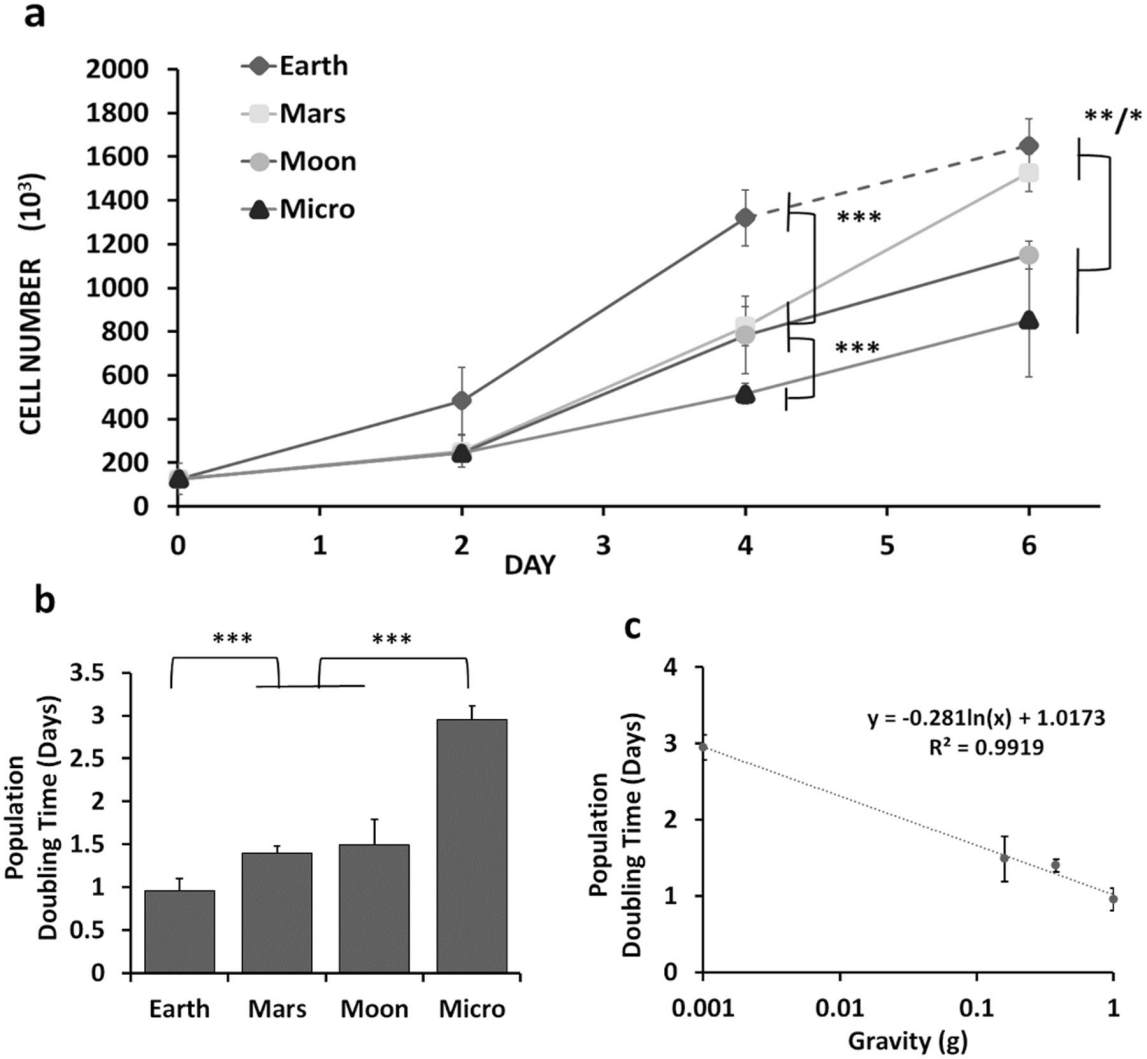
Inhibition of 7F2 cell proliferation in simulated partial gravity. **a** Increase in cell numbers on days 2 and 4 of culture in 1 G (Earth) and under the various altered simulated partial gravity conditions (Mars, Moon, Micro) (*N* = 3). **b** Specific partial population doubling times between days 2 and 4 and taking into account the mean absolute error (MAE). The average population doubling times were calculated as 0.96 ± 0.14 days for Earth (1 G), 1.4 ± 0.08 days for Mars, 1.5 ± 0.30 days for Moon, and 2.95 ± 0.17 days for simulated microgravity (Micro). **c** Semilogarithmic plot of the specific population doubling times vs. simulated partial gravity (*R*^2^ = 0.9919) for that 48-h window of time. Data are presented as means ± standard deviation. Asterisk (*) shows *p* < 0.05, (**) shows *p* < 0.01, (***) *p* < 0.001 as determined by Tukey’s post hoc analysis (panels (**a**) and (**c**)) or mean absolute error (MAE) (panel (**b**)).

Simulated microgravity, such as in the RPM (~10^−3^ × G)^12^, inhibits cell proliferation in numerous cell types, including in bone-marrow-derived adult stem cells (BMSCs)^36^. The proliferation of i7F2 preosteoblasts was significantly inhibited in a dose-dependent manner on days 4 and 6 for the simulated partial gravity (SPG) of Moon and Mars and simulated microgravity (Micro) (Fig. 1a). The time between days 2 and 4 represented a window in which the cells actively proliferated under all gravity conditions; between days 4 and 6 the cells actively proliferated under Mars and Moon conditions but reached confluence in Earth gravity. Cell seeding was weighed between being too low, prompting cell senescence and inhibiting layer formation, and being too high (rapid confluence). During the 2–4 day window for 7F2s in these conditions, we observed statistically significant differences in the population doubling times (PDTs) between Earth, Mars/Moon, and Micro (Fig. 1b). In Micro, the PDTs, specific for that 2–4 day time period, increased three-fold over Earth controls (2.95 ± 0.16 days (Micro) vs 0.96 ± 0.15 days (Earth)). The PDTs for Mars and Moon conditions were 1.4 ± 0.08 days and 1.5 ± 0.30 days, respectively (Fig. 1b). Compared to the specific PDT values of cells cultured in Earth gravity, exposure to Micro slowed proliferation and resulted in a relative increase in the doubling time by 307% (*P* < 0.001), by 156% in Moon (*P* < 0.001), and by 146% for Mars (*P* < 0.001) (MAE). A semilogarithmic plot of PDTs against nominal gravity values on Earth, Mars, Moon, and microgravity showed a very good fit (*R*^2^ = 0.9919) (Fig. 1c).

### Alkaline phosphatase enzymatic activity is inhibited by partial gravity

**Fig. 2:**
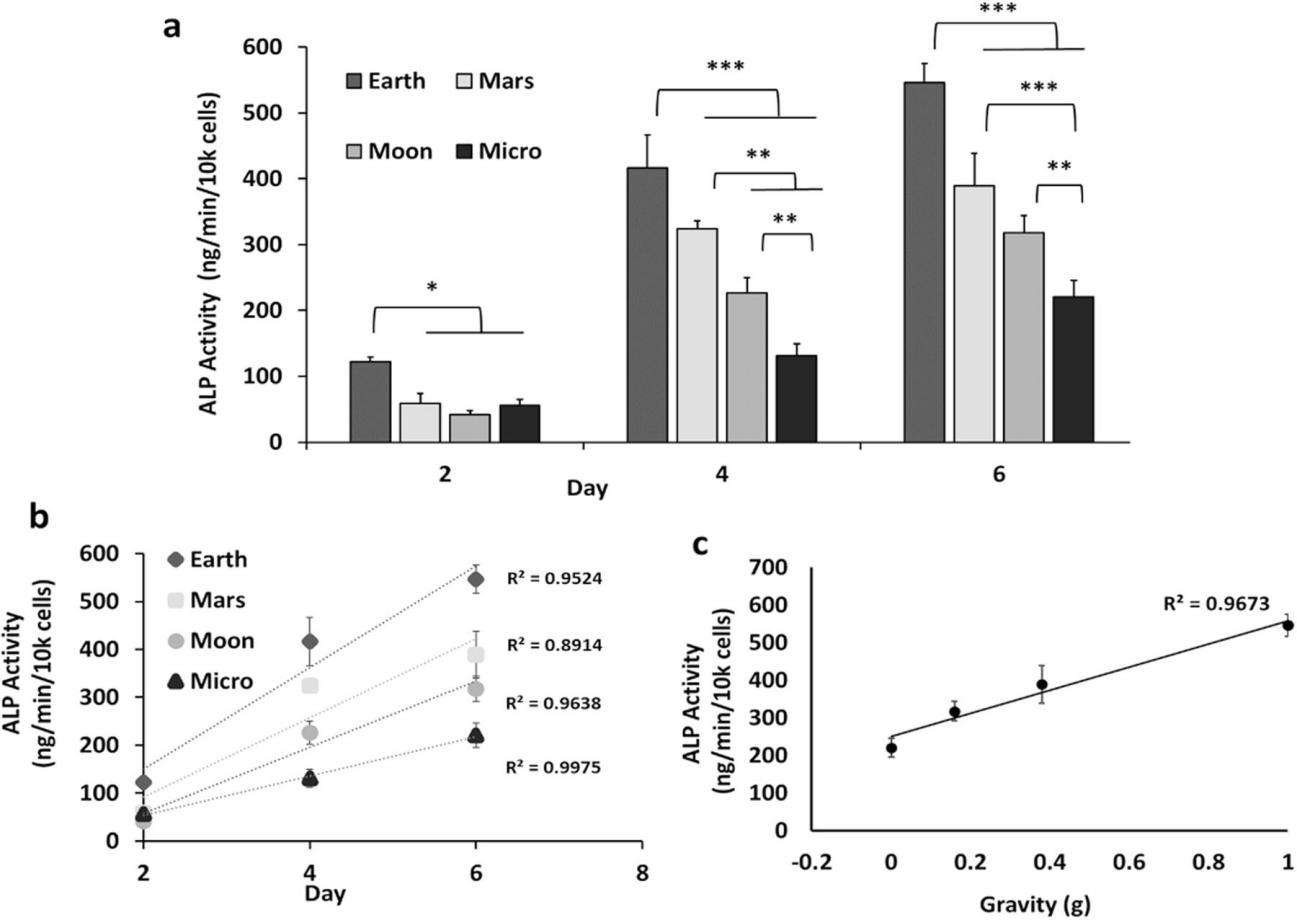
Simulated partial gravity inhibits osteogenic differentiation in 7F2 osteoblasts. **a** ALP activity as a result of exposure to different simulated partial-gravity conditions at different time points (*N* = 3). **b** ALP activity as a function of time between day 2 and day 6. **c** ALP activity as a function of altered simulated partial gravities at day 6. Data are presented as means ± standard deviation. Asterisk (*) shows *p* < 0.05, (**) shows *p* < 0.01, (***) *p* < 0.001 as determined by Tukey’s post hoc analysis.

Alkaline phosphatase is a widely used marker for osteoblast maturation and an important regulator of osteoblast mineralization^37–40^. To quantify the impairment of osteogenic differentiation by SPG, we measured ALP activity in 7F2 preosteoblasts, cultured for up to 6 days in osteogenic media in Earth (control) and SPG conditions (Mars, Moon, Micro). ALP activity was normalized to cell numbers as determined via the PICO-green assay. As seen in Fig. 2a, all simulated non-Earth gravities resulted in a time- and dose-dependent reduction in ALP activity in comparison to the Earth control. For example, on day 6, ALP activity was reduced by 27 ± 7% (*p* < 0.0001), 40 ± 5% (*p* < 0.0001) and 58 ± 9% (*p* < 0.0001) for Mars, Moon and Micro, respectively (Fig. 2b) (Tukey post hoc). Plotting ALP activity on day 6, the time point of maximal signal, against altered gravity levels gives a linear trend (Fig. 2c) with a correlation coefficient of 0.967.

### Simulated partial gravity downregulates the expression of osteogenic genes

**Fig. 3:**
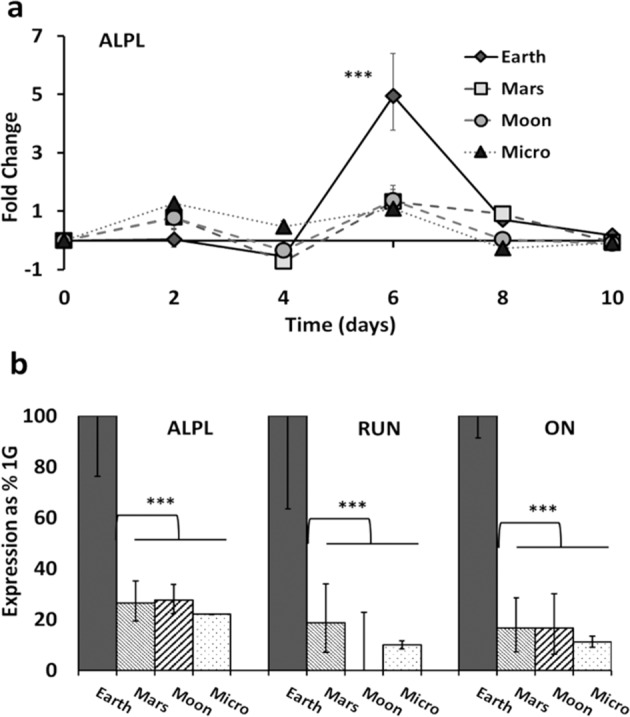
Effects of simulated partial gravities on osteogenic marker gene expression. **a** Time course of ALPL gene expression for multiple simulated gravity conditions, following exposure of confluent 7F2 osteoblasts to osteogenic media. Relative expression was analyzed by real-time PCR and normalized to the levels of a housekeeping gene (GAPDH), with 1 G confluent cell culture (prior to osteogenic induction) as control. The relative expression of each gene to control is presented as a fold-change expression for each transcript. **b** Inhibition by simulated partial gravity of the three osteogenic marker genes is best discernable at the peak of their expression at day-6 Alkaline phosphatase (ALPL), Runt-related Transcription Factor 2 (RUN), Sparc/osteonectin (ON). Data are presented as means ± standard deviation. Values are means ± SD of three independent cultures. Asterisk (*) shows *p* < 0.05, (**) shows *p* < 0.01, (***) *p* < 0.001 as determined by Tukey’s post hoc analysis.

Next, we evaluated the effects of the three SPG conditions (Mars, Moon, Micro) on the early expression of select osteogenic marker genes, Runt-related Transcription Factor 2 (RUN), Alkaline Phosphatase (ALPL), and Osteonectin (ON), using quantitative RT-PCR. Expression levels of these osteogenic markers were measured every 2 days for 10 days. Qualitatively, the pattern of expression of all these genes followed a similar time- and dose/gravity-dependent profile. For example, the transient expression of ALPL peaked at day 6 under 1 G conditions (Fig. 3a). At its peak, ALPL expression was downregulated under all levels of SPG, with no significant difference between Mars, Moon, or microgravity (Fig. 3b). By comparison to their expression levels on earth, all marker genes studied were suppressed by SPG. Specifically, on day 6, ALPL gene levels were reduced 3.86-fold in simulated microgravity, 3.58-fold under Moon conditions, and 3.63-fold under Mars conditions. Similarly, the expression of the RUN was reduced 2.93-fold (simulated microgravity), 3.3-fold (Moon), 2.65-fold (Mars), respectively, and ON by 3.43-fold (microgravity), 3.224-fold (Moon), 3.226-fold (Mars) (*p* < 0.0001) for all stated values) (Tukey post hoc). The expression levels of the individual genes at days 2, 6, 8, and 14 can be found in Supplementary Fig. 2 in the Appendix.

### Simulated partial-gravity conditions reduce long-term mineralization

**Fig. 4:**
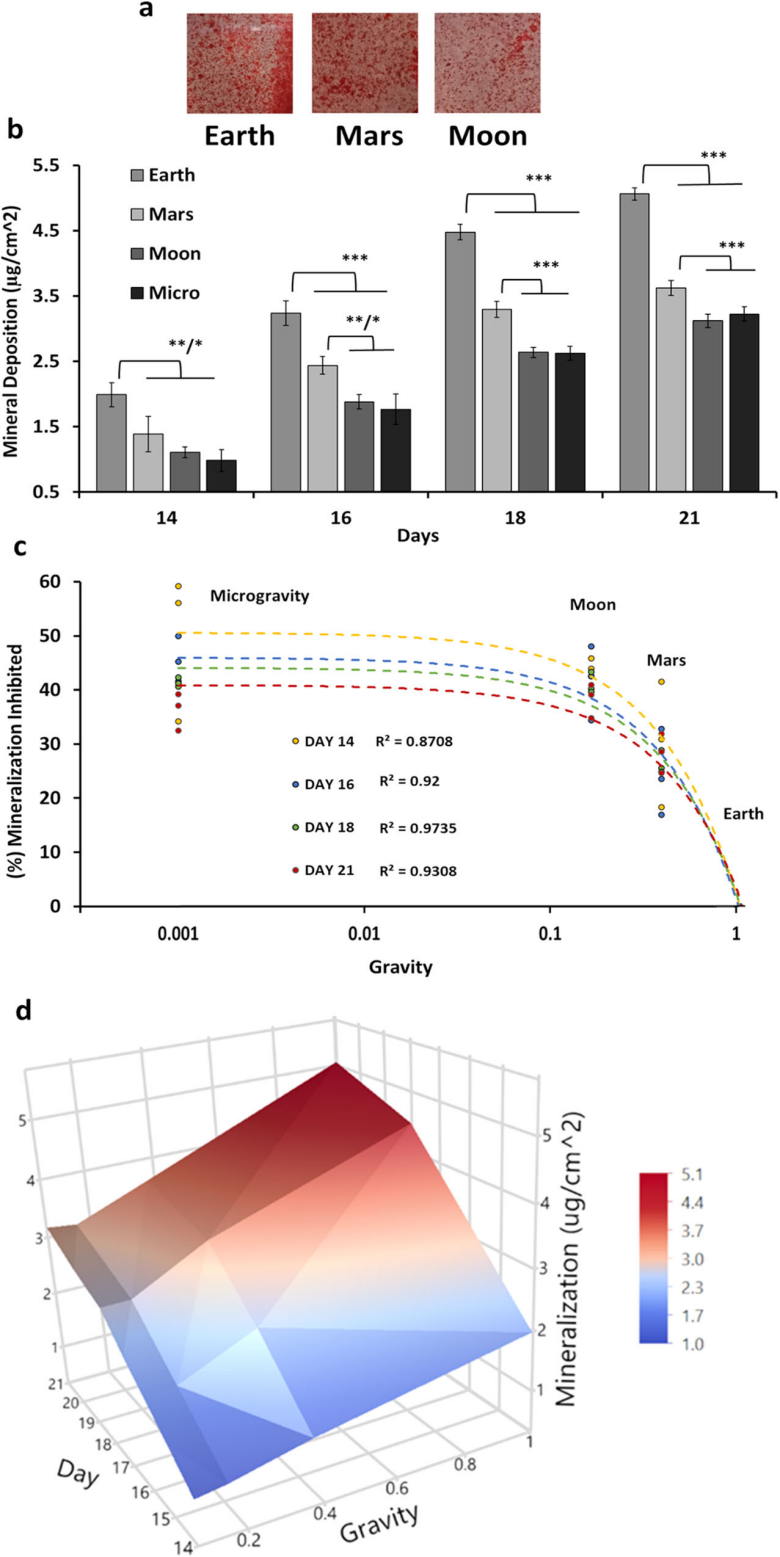
Effect of simulated partial gravity on long-term mineralization. **a** Alizarin red-stained mineralized nodules after 18 days on Earth (1 G) and in the RPM exposed to simulated partial -gravities of Moon and Mars. Images are representative micrographs of 1 × 1 cm^2^ areas from the bottom of T-12.5 Falcon™ Tissue Culture Treated Flasks. **b** Long-term mineralization under variable simulated gravity conditions, quantified as micrograms of calcium per square centimeter (µg/cm^2^). **c** Percentage inhibition of mineralization normalized versus Earth controls with modeled trendlines for various days: 14 days (*R*² = 0.8708), 16 days (*R*² = 0.92), 18 days (*R*² = 0.9735), 21 days (*R*² = 0.9308). **d** Three-dimensional surface plot and heatmap of the interplay between simulated gravity levels and mineralization over time. Data are presented as means ± standard deviation (*N* = 3). Asterisk (*) shows *p* < 0.05, (**) shows *p* < 0.01, (***) *p* < 0.001 as determined by Tukey’s post hoc analysis.

Mineral nodule formation was initially assessed qualitatively by visual inspection following alizarin red staining (Fig. 4a). Quantitative analysis of the differences in mineralization between the various simulated partial-gravity culture conditions (Mars, Moon, Micro) was assessed using the TECO mineralization assay. Differences in calcium deposition between the various simulated gravities became first noticeable by day 14 of RPM culture and were studied through day 21 (Fig. 4b). Relative to the 1 G controls, mineralization was significantly decreased by all SPG conditions. Inhibition due to Mars gravity was significantly different from Moon and Micro (*P* < 0.001, Tukey post hoc), while Moon and Micro were not significantly different from each other.

Percentage inhibition was calculated with 1 G (Earth) as the control. Five models were constructed to best fit the data for various days of SPG (plus Earth), and also for an aggregate of all days (*R*^2^ = 0.8972). SPG inhibited osteoblast mineralization along a linear gradient, with mineralization under Mars conditions being significantly different from that under Moon or Micro conditions, as illustrated in (Fig. 4c). The effects of simulated altered gravity over time on mineralization are shown in a three-dimensional surface plot and heatmap (Fig. 4d). The surface plot was derived from Equation (1), where (*t*) is time (days), (*g*) is gravity, and (mineral) is mineralization in µg/cm^2^.

1 $$({{{\mathrm{mineral}}}}) = \left( { - 3.87} \right) + 0.34 \ast \left( t \right) + 1.63 \ast \left( g \right) + \left( {\left( t \right) - 0.38525} \right) \ast \left( {\left( {\left( g \right) - 17.25} \right) \ast 0.14} \right)$$

## Discussion

The most significant and novel finding in this work is the uncovering of differential partial-gravity dependencies of the parameters studied: dose dependence versus step-function. While most ground-based studies using the RWV or the RPM bioreactors have focused on the effects of “simulated microgravity” (Micro, ~10^−3^ G), there are a few studies^25,31^ that compare the effects of Micro and non-terrestrial simulated partial gravities (Moon, Mars). Given the preparations for upcoming manned spaceflights to Moon and Mars, there is a need for increased studies of partial-gravity research. Amongst the research priorities between 2018 and 2024, recent NASA workshops and international committees have emphasized studies of “G-dose response in cell cultures using RPM” on Earth, as well as ISS-based counter-experiments^41^.

In the past, earth-based simulation of partial gravity has been challenging. Some of the previous approaches for generating micro and/or partial gravity, such as drop towers (seconds of weightlessness), parabolic flights (~10–20 s), sounding rockets (3–8 min in free-fall phase), and commercial sub-orbital rockets (minutes to hours) have significant drawbacks and pitfalls, such as short duration and fluctuation of the gravitational load and intrinsic experimental challenges^42^. The system we used to simulate the extraterrestrial partial gravities of Moon and Mars in addition to simulating microgravity, the new software-driven RPM^SW^, has been described previously^25,31,43^ The RPM^SW^ is the advanced version of the original “Random Positioning Machine”, which has been widely used to simulate microgravity^19,20^. However, instead of leveraging the random motion of the original RPM, the RPM^SW^ employs specific RPM path files, as described in great detail in the Supplementary Information^25^ of Manzano et al., briefly explained in the “Materials and methods” section of this paper, and also illustrated in the Appendix (Supplementary Figs. 3 and 4). To the best of our knowledge only the RPM^SW^ (and similar devices), or a centrifuge in orbit can provide long-term simulated partial gravity of this quality and duration.

The similarities between our results and those of others using clinostats as earth-based analogs for simulating microgravity and partial-gravity conditions and results observed in space-flown sample in real microgravity, suggest that many of the reported effects might primarily be due to alterations in the modeled reduced gravity conditions. However, for the sake of scientific rigor, and as the mechanisms of gravisensing in SMG or SPG are as yet unclear^44^ we would like to acknowledge the possibility that some of the phenomena observed in clinostats and the RPM could also be (in part) due to indirect effects in the environment (e.g., changes in fluid shear or mass transfer)^44–47^. Moreover, while differences in vibration, fluid shear, or mass transfer may occur between different path files in the RPM^SW^, these have yet to be quantified or identified as significant^25,31^.

The gravity-dependent, graded inhibition of cell proliferation and ALP activity (Figs. 1 and 2) can be taken as an indirect validation of the ability of our system to simulate different magnitudes of partial gravity. Our identification of graded, step, and threshold inhibition due to partial gravitational unloading has some precedence: for example, using the RPM to simulate partial gravity, Kamal et al. observed a gradient in morphofunctional types of nucleoli between Earth, Mars, and Micro after 24 h as well as in Histone H4 acetylation^31^. Manzano et al. reported that cell proliferation of *Arabidopsis thaliana* plants at simulated Mars gravity was essentially identical to that on Earth, while it was significantly accelerated under Micro conditions, with Moon inhibition being between Mars and Micro^25^. The authors speculate that there is a likely threshold to gravity sensitivity intermediate between Moon and Mars. Swift et al. used a weight-bearing suspension device to investigate musculoskeletal losses following full and partial hindlimb unloading, akin to modeled partial gravity on Moon, Mars, and in simulated microgravity^48^. Close inspection of the data indicated different graded responses depending on the anatomical location of the individual plantar-flexor muscles. For example, loss of vBMD (bone mineral density) of the proximal tibia was similar for Moon and Mars, just as we found for our data on ALP activity and osteoblast proliferation (Figs. 1 and 2). While different from our system in the details, the results of these studies do show both gradients and thresholds in terms of gravity dependence, just like our data.

Our findings confirm the results of previous studies indicating the inhibitory effect of SMG on osteoblast function^12,17,33,36,49–51^. In expanding those studies, our data indicate a significant inhibitory effect of simulated partial gravity (SPG) on both proliferation, enzymatic activity, and gene expression in the short-term experiments (6 days), as well as on mineralization in the long-term studies (21 days). From our results we found three different patterns of inhibition: (*Pattern I*) in this pattern, inhibition of proliferation between days 2 and 4 and enzymatic activity by Moon and Mars gravity levels were statistically indistinguishable (Figs. 1b and 2a). A different gradient was observed in inhibition of mineralization (*Pattern II*) that displayed similarity between Moon and Micro (Fig. 4c). By contrast to the functional studies, inhibition of the expression of the three osteogenic marker genes studied exhibited a threshold effect (*Pattern III*, Fig. 3): gene expression levels in cells exposed to Mars, Moon, and Micro were all similar to each other but significantly different from gene expression in 1 G. ALP activity illustrates the contrast: inhibition of ALP-enzymatic activity was greater in Micro than on Mars, while expression of the ALPL gene was similar between Mars, Moon, and Micro (Fig. 3b). To the best of our knowledge, this study provides a first comparative analysis of the effects of non-terrestrial gravities (Mars, Moon) versus Earth and microgravity conditions on cultured mammalian cells. Based on the predictive character of our mineralization model (Fig. 4e), we can make predictions about conditions based on other gravity wells, like Europa (0.134 G).

Our in vitro results are in line with previous space-based studies in orbital microgravity. For example, using the osteosarcoma MG-63 cell line, Carmeliet et al. examined the effects of “real” microgravity in space on matrix formation and maturation, both at the protein and mRNA levels. Cells were cultured for 9 days under orbital microgravity conditions aboard a Foton-10 satellite. Alkaline phosphatase activity in microgravity increased by only a factor of 1.8 over the culture period, compared to the 3.8-fold increase in the ground-based 1 g controls (*p* < 0.01). Similarly, gene expression for ALPL in microgravity was decreased, producing a fold change of 0.6 (*p* < 0.02)^51^. These results are qualitatively similar to our results in simulated microgravity: we observed a 2.5-fold reduction of ALP-enzymatic activity between Micro and Earth and a fold change of 0.8 in ALPL gene expression (Fig. 3).

While ALP activity plays a key role in the mineralization and maintenance of bone, the exact mechanisms of action are unclear^49^. Sugawara et al. demonstrated that the enzymatic activity of ALP is necessary for mineralization in MC3T3 cells, but does not require anchoring of ALP to the external surfaces of plasma membranes, as ALP is released by osteoblasts and steadily accumulates in the media^52^. Elevated levels of ALP do not necessarily correlate with increased mineralization; for example, mechanical stimulation of osteoblasts via pulsatile flow elicited an increase in ALP activity but did not result in a significant difference in matrix mineralization^53^. The decrease in the expression and activity of alkaline phosphatase and the inhibition of genes related to matrix proteins, like osteopontin and osteocalcin, which are characteristic of mature mineralizing osteoblasts, can indicate that osteogenic cells are sensitive to mechanical stimuli—in our case partial gravity—in both their mature and immature phases.

While one study found no effects of SMG on the proliferation of 2T3 cells cultured in the RPM^24^, most others have observed a significant inhibition of cell proliferation. For example, Dai et al. reported that the proliferation of rat bone-marrow mesenchymal stem cells was inhibited by SMG, with cells arrested in the G0/G1 phase of the cell cycle^36^. Similarly, skeletal unloading, a ground-based analog to SMG, has been shown to decrease the proliferation of osteoblast precursor cells^54^. Our study suggests, for the first time, a dose-dependent reduction of proliferation in osteoblasts by simulated partial gravity (Fig. 1). A plot of the calculated doubling times vs the distinct gravity levels suggests a logarithmic relationship (Fig. 1c). By contrast, the gravity dependence of the inhibition of enzymatic ALP activity (Fig. 2) or matrix mineralization (Fig. 4) appears to be linear.

There is a consensus that ALPL mRNA levels are inhibited by microgravity over time, though there have been exceptions observed. Many studies have observed downregulation of ALPL in both real and simulated microgravity versus Earth controls^24,33,55,56^, for example in 2T3 cells after 3 days in the RPM^19^. A smaller number of studies have reported ALPL upregulation in microgravity versus Earth gravity, for example in cultured osteoblasts recovered after 10 days in space^14,57,58^ or in rats that were flown in space for 6 days^43^. Explaining this upregulation, Kapitonova et al., speculated that this unexpected upregulation may be due to the change in the window of matrix maturation as microgravity inhibits osteoblasts. As noted by Landis et al. preosteoblasts cultured in space experienced a delayed progression into a mature mineralizing state, as microgravity reduced the expression of osteocalcin and type-I collagen^59^. Finally, at least one study that lasted one day only showed no difference in ALPL expression^16^. Our studies confirm and build on the majority of prior publications indicating that microgravity inhibited the initiation of mineralization in osteoblasts and downregulated genes related to osteoblast mineralization (Fig. 3). Another example for a similar outcome is MC3T3-E1 cells in Hu et al.^17^.

In addition to ALPL, our study also focused on Runx2 and osteonectin. Runx2 (or RUN), a member of the runt homology domain transcription factor family, is a transcription factor essential for osteoblast differentiation and mineralization^16^, and for regulating osteocalcin expression^24^. Decreased expression of ALPL and RUN is consistently observed in most orbital experiments and SMG cultures using calvaria, MSCs, and cell lines^17,24,60^. Peak RUN expression occurs at the end of the proliferative phase and the beginning of mature mineralization and matrix deposition. ALPL and RUN are often profiled in parallel. For example, Pardo et al., while finding no change in 2T3 cell proliferation, reported that after 3 days of culture in the RPM under Micro conditions, ALPL gene expression was reduced five-fold while RUN was reduced 1.88-fold when compared to 1 G Earth controls^24^. Hu et al. observed a significant (~2-fold) reduction in the expression of the genes for ALPL and RUN in 7-day differentiated MC3T3 cells after 24 h in SMG^17^. By comparison, the expression of the osteogenic genes in our experiments was downregulated (day 6, Micro) as follows: ALPL was inhibited 3.85-fold and RUN 2.93-fold.

In vitro confluence and osteogenic differentiation resulted in the downregulation of proliferation and the transient expression of ALPL and RunX2. Both these genes are associated with osteogenic differentiation, reaching a maximal expression on day 6, similar to the expression profile in Choi et al., using periodontal ligament cells^61^, or Bikle et al. in hindlimb elevation^58^, or Guillot et al. in adult bone-marrow MSCs^62^. The maximal expression followed by a decline may indicate the initiation of differentiation and the transition of a majority of the cells from a proliferating to a more mature matrix-mineralizing phenotype, making this time point (or transition point) of particular interest when investigating either the inhibition or delay of differentiation. Stein et al. refer to this pattern as “stage-specific” or transient expression^28^.

Osteonectin (ON), also known as secreted protein acidic and rich in cysteine (SPARC), is one of the most abundant non-collagenous bone proteins^63^. The effects of (simulated) microgravity on ON expression are controversial. While some studies show a significant reduction of ON expression in cells exposed to Micro conditions^64^, just as we have demonstrated in this work (Fig. 3b), others have seen no significant reduction in expression due to microgravity^14,57^. By contrast, Kumei et al. reported a small increase in osteonectin under microgravity (space shuttle) conditions^65^, while Kapitonova et al. reported a non-significant difference^57^. Our results (Fig. 3a) demonstrate a transient peak expression of osteogenic genes (e.g., ALPL, RANKL, BMP-4, Collagen-I, osteocalcin), a profile seen previously in other mineralizing cells, like periodontal cells differentiating into osteoblasts^61^.

Buravkova et al. have speculated that these mechanically sensitive markers of osteoblast differentiation are most vulnerable to changes in the gravitational field at the peak of expression^49^. Importantly, we did not observe statistically significant differences in gene expression between the simulated partial gravities: Mars, Moon, and microgravity. Thus, we presume that the inhibition of gene expression exhibits a threshold or step-function behavior, with the threshold itself somewhere between Mars gravity (0.38 G) and Earth (1 G). This is different from the dose-responses observed in differentiation, mineralization, or proliferation and novel in terms of observation of osteoblast functionality and bone health. The threshold between Earth and Mars in terms of modulation of gene expression by SPG suggests a sensitivity of gene expression to the initial changes in gravitational load not shared by other processes.

There is an intense interest in space biology regarding thresholding, particularly for gravity sensing^42^ and for biological impairment. Studies at simulated partial gravity can contribute to the determination of thresholds for various biological responses to gravitational alterations. For example, the effects of partial gravity on critical physiological factors like the cell cycle are understudied^31^. It is important to determine how exposure to distinct magnitudes of gravity interacts with time (effects after 6 h, 24 h, weeks). Other papers, like Kamal et al. have, in plants, observed threshold effects related to exposure time and, for example, ribosome biogenesis, where the effects are similarly inhibited by simulated Mars gravity and microgravity in comparison to Earth gravity, suggesting a threshold between 1 g and 0.38 g^31^, similar to our finding for the inhibition of gene expression in 7F2 preosteoblasts (Fig. 3b).

While there are several hypotheses for gravity sensing mechanisms, epigenetic triggers, and potential feedback loops in mammalian cells, that exacerbate inhibition over time^66^, the dependence of these mechanisms across a range of simulated gravities has not yet been elucidated and warrants further investigation in future spaceflights to Moon and Mars. In the meantime, a reasonable validation of these results, and other partial-gravity simulations on Earth, could also be done in orbital microgravity on the ISS, using a variable counterweight centrifuge^42^. In the past, centrifuges have been tested in space to simulate Earth gravity, though challenges persist in replicating methodologies and carrying out cell culture experiments that require intensive intervention or attention from astronauts.

Taken together, exposure of 7F2 osteoblasts to various levels of simulated partial gravity (equivalent to ~0.6–10^−3^ G), resulted in significant, gravity-dependent inhibition of ALP activity in the short-term (6 days) and of mineralization in the long term (21 days). Proliferation was also inhibited, with decreasing gravity significantly lengthening population doubling times. Gradients for inhibition exhibited slightly different patterns for the various parameters studied: the pattern of inhibition of cell proliferation and ALP activity was 1 G > Mars=Moon>Micro, while the pattern of inhibition of matrix mineralization was 1 G > Mars>Moon=Micro. Modulation of gene expression by reductions in simulated gravity levels exhibited a threshold-like behavior, with all partial-gravity states and Micro resulting in similar levels of downregulation. Describing, for the first time, dose-dependent osteogenic responses to reductions in simulated partial gravity, our comparative study is timely and relevant for forthcoming space explorations, specifically for predicting biological effects of the reduced gravity on Moon and Mars.

## Supplementary information

Supplementary Figures and Notes

Reporting Summary

## Data Availability

The data of this study are available from the authors upon reasonable request.

## References

[1] LeBlanc AD, Spector ER, Evans HJ, Sibonga JD (2007). Skeletal responses to space flight and the bed rest analog: a review. J. Musculoskelet. Neuronal Interact..

[2] Lang T (2004). Cortical and trabecular bone mineral loss from the spine and hip in long-duration spaceflight. J. Bone Min. Res..

[3] Zerath E (2000). Spaceflight inhibits bone formation independent of corticosteroid status in growing rats. J. Bone Miner. Res..

[4] Nelson ES, Jules K (2004). The microgravity environment for experiments on the International Space Station. J. Gravit. Physiol..

[5] Zerath E (1996). Effects of spaceflight and recovery on rat humeri and vertebrae: histological and cell culture studies. J. Appl Physiol..

[6] Monticone M, Liu Y, Pujic N, Cancedda R (2010). Activation of nervous system development genes in bone marrow derived mesenchymal stem cells following spaceflight exposure. J. Cell Biochem..

[7] Hughes-Fulford M (2001). Changes in gene expression and signal transduction in microgravity. J. Gravit. Physiol..

[8] Nabavi N, Khandani A, Camirand A, Harrison RE (2011). Effects of microgravity on osteoclast bone resorption and osteoblast cytoskeletal organization and adhesion. Bone.

[9] Tamma R (2009). Microgravity during spaceflight directly affects in vitro osteoclastogenesis and bone resorption. FASEB J..

[10] Orwoll ES (2013). Skeletal health in long-duration astronauts: nature, assessment, and management recommendations from the NASA Bone Summit. J. Bone Min. Res..

[11] Stavnichuk M, Mikolajewicz N, Corlett T, Morris M, Komarova SV (2020). A systematic review and meta-analysis of bone loss in space travelers. NPJ Microgravity.

[12] Makihira S, Kawahara Y, Yuge L, Mine Y, Nikawa H (2008). Impact of the microgravity environment in a 3-dimensional clinostat on osteoblast-and osteoclast-like cells. Cell Biol. Int..

[13] Kim YJ (2017). Time-averaged simulated microgravity (taSMG) inhibits proliferation of lymphoma cells, L-540 and HDLM-2, using a 3D clinostat. Biomed. Eng. Online.

[14] Rucci N, Migliaccio S, Zani BM, Taranta A, Teti A (2002). Characterization of the osteoblast-like cell phenotype under microgravity conditions in the NASA-approved Rotating Wall Vessel bioreactor (RWV). J. Cell Biochem..

[15] Saxena R, Pan G, McDonald JM (2007). Osteoblast and osteoclast differentiation in modeled microgravity. Ann. N. Y Acad. Sci..

[16] Chatziravdeli V, Katsaras GN, Lambrou GI (2019). Gene expression in osteoblasts and osteoclasts under microgravity conditions: a systematic review. Curr. Genomics.

[17] Hu LF, Li JB, Qian AR, Wang F, Shang P (2015). Mineralization initiation of MC3T3-E1 preosteoblast is suppressed under simulated microgravity condition. Cell Biol. Int..

[18] Poon C (2020). Factors implicating the validity and interpretation of mechanobiology studies in simulated microgravity environments. Eng. Rep..

[19] Grimm D (2014). Growing tissues in real and simulated microgravity: new methods for tissue engineering. Tissue Eng. Part B Rev..

[20] Wuest SL, Richard S, Kopp S, Grimm D, Egli M (2015). Simulated microgravity: critical review on the use of random positioning machines for mammalian cell culture. Biomed. Res. Int..

[21] Sato A (1999). Effects of microgravity on c-fos gene expression in osteoblast-like MC3T3-E1 cells. Adv. Space Res..

[22] Sarkar D (2000). Culture in vector-averaged gravity under clinostat rotation results in apoptosis of osteoblastic ROS 17/2.8 cells. J. Bone Min. Res..

[23] Gershovich P, Gershovich J, Zhambalova A, Romanov YA, Buravkova L (2012). Cytoskeletal proteins and stem cell markers gene expression in human bone marrow mesenchymal stromal cells after different periods of simulated microgravity. Acta Astronautica.

[24] Pardo SJ (2005). Simulated microgravity using the Random Positioning Machine inhibits differentiation and alters gene expression profiles of 2T3 preosteoblasts. Am. J. Physiol.-Cell Physiol..

[25] Manzano A (2018). Novel, Moon and Mars, partial gravity simulation paradigms and their effects on the balance between cell growth and cell proliferation during early plant development. NPJ Microgravity.

[26] Borst A, van Loon JJ (2009). Technology and developments for the random positioning machine, RPM. Microgravity Sci. Technol..

[27] Van Loon JJ (2007). Some history and use of the random positioning machine, RPM, in gravity related research. Adv. Space Res..

[28] Stein GS (2004). Runx2 control of organization, assembly and activity of the regulatory machinery for skeletal gene expression. Oncogene.

[29] Phelan MA, Gianforcaro AL, Gerstenhaber JA, Lelkes PI (2019). An air bubble-isolating rotating wall vessel bioreactor for improved spheroid/organoid formation. Tissue Eng. Part C. Methods.

[30] Benavides Damm T, Walther I, Wüest SL, Sekler J, Egli M (2014). Cell cultivation under different gravitational loads using a novel random positioning incubator. Biotechnol. Bioeng..

[31] Kamal KY, Herranz R, van Loon JJWA, Medina FJ (2018). Simulated microgravity, Mars gravity, and 2g hypergravity affect cell cycle regulation, ribosome biogenesis, and epigenetics in Arabidopsis cell cultures. Sci. Rep..

[32] Lin HY, Lin YJ (2011). In vitro effects of low frequency electromagnetic fields on osteoblast proliferation and maturation in an inflammatory environment. Bioelectromagnetics.

[33] Bucaro MA (2007). The effect of simulated microgravity on osteoblasts is independent of the induction of apoptosis. J. Cell Biochem..

[34] Du Y (2019). Topographic cues of a novel bilayered scaffold modulate dental pulp stem cells differentiation by regulating YAP signalling through cytoskeleton adjustments. Cell Prolif..

[35] Livak KJ, Schmittgen TD (2001). Analysis of relative gene expression data using real-time quantitative PCR and the 2(-Delta Delta C(T)) Method. Methods.

[36] Dai ZQ, Wang R, Ling SK, Wan YM, Li YH (2007). Simulated microgravity inhibits the proliferation and osteogenesis of rat bone marrow mesenchymal stem cells. Cell Prolif..

[37] Orimo H, Shimada T (2008). The role of tissue-nonspecific alkaline phosphatase in the phosphate-induced activation of alkaline phosphatase and mineralization in SaOS-2 human osteoblast-like cells. Mol. Cell Biochem..

[38] Orimo H (2010). The mechanism of mineralization and the role of alkaline phosphatase in health and disease. J. Nippon Med. Sch..

[39] Anderson HC (1995). Molecular biology of matrix vesicles. Clin. Orthop. Relat. Res..

[40] Wennberg C (2000). Functional characterization of osteoblasts and osteoclasts from alkaline phosphatase knockout mice. J. Bone Min. Res..

[41] Clément G (2017). International roadmap for artificial gravity research. NPJ Microgravity.

[42] Kiss JZ, Wolverton C, Wyatt SE, Hasenstein KH, van Loon JJWA (2019). Comparison of microgravity analogs to spaceflight in studies of plant growth and development. Front. Plant Sci..

[43] Herranz R, Valbuena MA, Manzano A, Kamal KY, Medina FJ (2015). Use of microgravity simulators for plant biological studies. Methods Mol. Biol..

[44] Grimm D (2022). The fight against cancer by microgravity: the multicellular spheroid as a metastasis model. Int. J. Mol. Sci..

[45] Klaus DM (2001). Clinostats and bioreactors. Gravit. Space Biol. Bull..

[46] Nickerson CA (2003). Low-shear modeled microgravity: a global environmental regulatory signal affecting bacterial gene expression, physiology, and pathogenesis. J. Microbiol. Methods.

[47] Yang J, Barrila J, Roland KL, Ott CM, Nickerson CA (2016). Physiological fluid shear alters the virulence potential of invasive multidrug-resistant non-typhoidal. NPJ Microgravity.

[48] Swift JM (2013). Partial weight bearing does not prevent musculoskeletal losses associated with disuse. Med. Sci. Sports Exerc..

[49] Buravkova LB, Gershovich PM, Gershovich JG, Grigor’ev AI (2010). Mechanisms of gravitational sensitivity of osteogenic precursor cells. Acta Nat..

[50] Capulli M, Rufo A, Teti A, Rucci N (2009). Global transcriptome analysis in mouse calvarial osteoblasts highlights sets of genes regulated by modeled microgravity and identifies a “mechanoresponsive osteoblast gene signature”. J. Cell Biochem..

[51] Carmeliet G, Nys G, Stockmans I, Bouillon R (1998). Gene expression related to the differentiation of osteoblastic cells is altered by microgravity. Bone.

[52] Sugawara Y, Suzuki K, Koshikawa M, Ando M, Iida J (2002). Necessity of enzymatic activity of alkaline phosphatase for mineralization of osteoblastic cells. Jpn. J. Pharm..

[53] Nauman EA, Satcher RL, Keaveny TM, Halloran BP, Bikle DD (2001). Osteoblasts respond to pulsatile fluid flow with short-term increases in PGE(2) but no change in mineralization. J. Appl Physiol..

[54] Machwate M (1993). Skeletal unloading in rat decreases proliferation of rat bone and marrow-derived osteoblastic cells. Am. J. Physiol..

[55] Bucaro MA (2004). Bone cell survival in microgravity: evidence that modeled microgravity increases osteoblast sensitivity to apoptogens. Ann. N. Y Acad. Sci..

[56] Patel MJ (2007). Identification of mechanosensitive genes in osteoblasts by comparative microarray studies using the rotating wall vessel and the random positioning machine. J. Cell Biochem..

[57] Kapitonova MY (2013). Alteration of cell cytoskeleton and functions of cell recovery of normal human osteoblast cells caused by factors associated with real space flight. Malays. J. Pathol..

[58] Bikle DD, Harris J, Halloran BP, Morey-Holton E (1994). Altered skeletal pattern of gene expression in response to spaceflight and hindlimb elevation. Am. J. Physiol..

[59] Landis WJ, Hodgens KJ, Block D, Toma CD, Gerstenfeld LC (2000). Spaceflight effects on cultured embryonic chick bone cells. J. Bone Min. Res..

[60] Shi W (2017). Microgravity induces inhibition of osteoblastic differentiation and mineralization through abrogating primary cilia. Sci. Rep..

[61] Choi MH, Noh WC, Park JW, Lee JM, Suh JY (2011). Gene expression pattern during osteogenic differentiation of human periodontal ligament cells in vitro. J. Periodontal Implant Sci..

[62] Guillot PV (2008). Comparative osteogenic transcription profiling of various fetal and adult mesenchymal stem cell sources. Differentiation.

[63] Rosset EM, Bradshaw AD (2016). SPARC/osteonectin in mineralized tissue. Matrix Biol..

[64] Zayzafoon M, Gathings WE, McDonald JM (2004). Modeled microgravity inhibits osteogenic differentiation of human mesenchymal stem cells and increases adipogenesis. Endocrinology.

[65] Kumei Y (2006). Microgravity signal ensnarls cell adhesion, cytoskeleton, and matrix proteins of rat osteoblasts: osteopontin, CD44, osteonectin, and alpha-tubulin. Ann. N. Y Acad. Sci..

[66] Takahashi K (2021). Gravity sensing in plant and animal cells. NPJ Microgravity.

