# Supplementary material for: Impairment of 7F2 osteoblast function by simulated partial gravity in a Random Positioning Machine
Source: NPJ Microgravity. 2022 Jun 7;8:20. doi: 10.1038/s41526-022-00202-x (PMC9174291; doi:10.1038/s41526-022-00202-x)
Supplement: Supplementary file 1 — Supplementary Figures and Notes [file 41526_2022_202_MOESM1_ESM.pdf]

Supplementary Information for "Impairment of 7F2 Osteoblast Function by Simulated Partial Gravity in a Random Positioning Machine."

Justin Braveboy-Wagner and Peter I. Lelkes

Supplementary Notes:

RPM Partial Gravity Precedent. Precedent for the use of the RPM to simulate partial gravities via path-file can be found in Manzano *et al*, which utilized the same path-files provided by Airbus, as used in this study. Each path file contains roughly 345,000 lines of code corresponding to the positional movements of the RPM frames over 24 hours and can be set to repeat. In addition to the supplementary information provided by Airbus for Manzano, a summary of the motion-modes for the RPM is outlined in **Supplementary Figure 3**. Additionally, mathematical verification of the vector average of the path files is provided by software that measures, in real-time, the frame movements of the RPM itself. This software is also necessary to detect when or if errors occur, such as the arm getting caught, stuck, tangled, or a mechanical failure like a band breaking due to age and overuse. **Supplementary Figure 4** displays mathematical outcomes for various modes, specifically the vector averages for path files and the random mode across 15 minutes of running time.

Supplementary Figures:

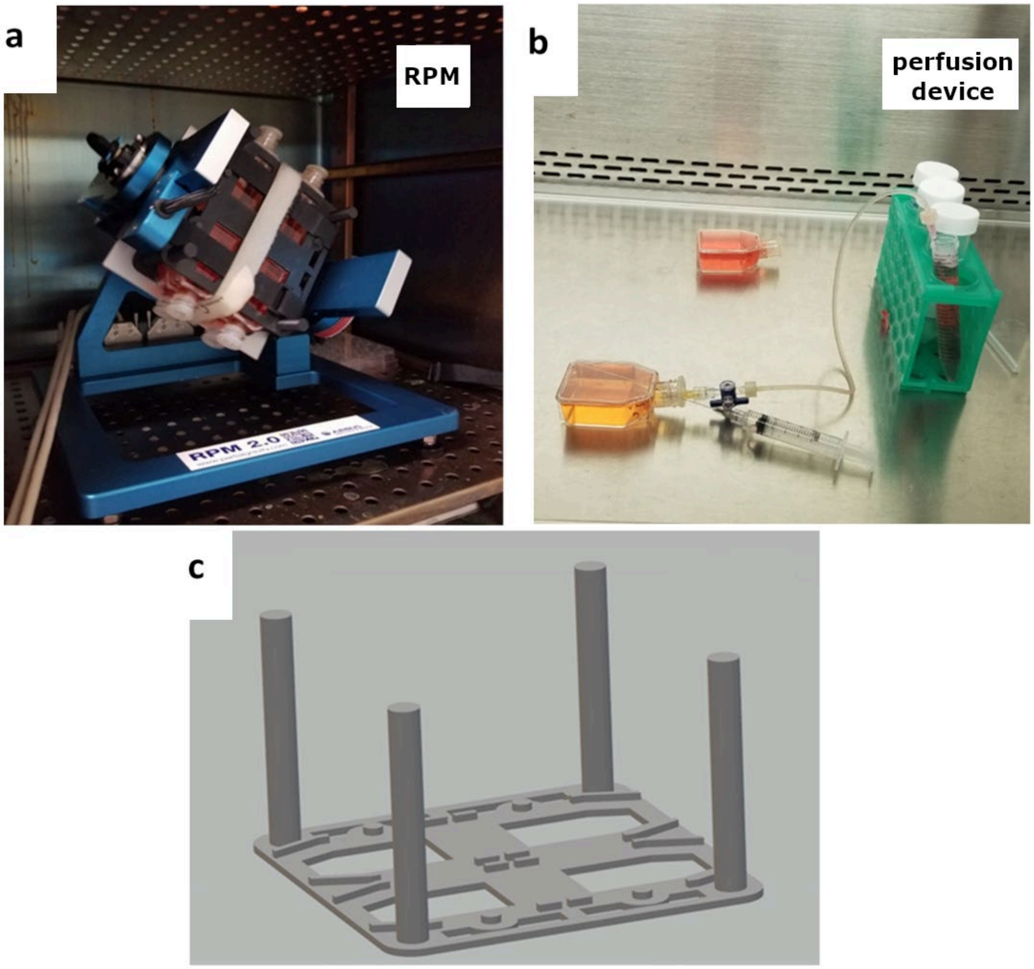

Supplementary Figure 1. RPM Cell Culture Accessories. To be able to use the RPM over days and weeks required the in-house development and fabrication of several accessories. **(a)** 3D printed cage for rapid loading and unloading (mounting and unmounting) of T-12 flasks. Velcro straps were used in combination with the 3-D cage to keep flasks secured but not entangled. Media changes of caged flasks could be concluded within ten minutes, extracting the cells in pairs after briefly pausing the clinostat, thus minimizing the time cells spent outside experimental specific partial gravity. **(b)** Perfusion system that was used to remove bubbles from flasks sealed with a gas-permeable silicone septum cap. The negative pressure of the syringe and needle aided in removing the bubble drew in fresh media. **(c)** 3D CAD model of the flask cage.

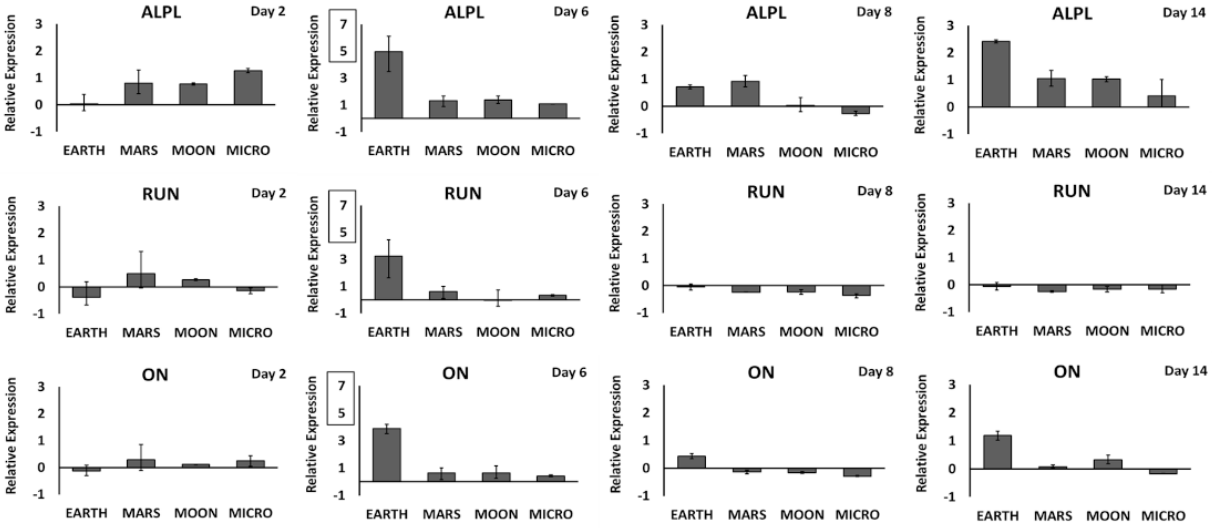

Supplementary Figure 2. Relative mRNA expression of osteogenic genes (ALP RUN and ON) in response to partial gravity, across multiple select days, up to day-14. Values are Means +/- SD of three cultures.

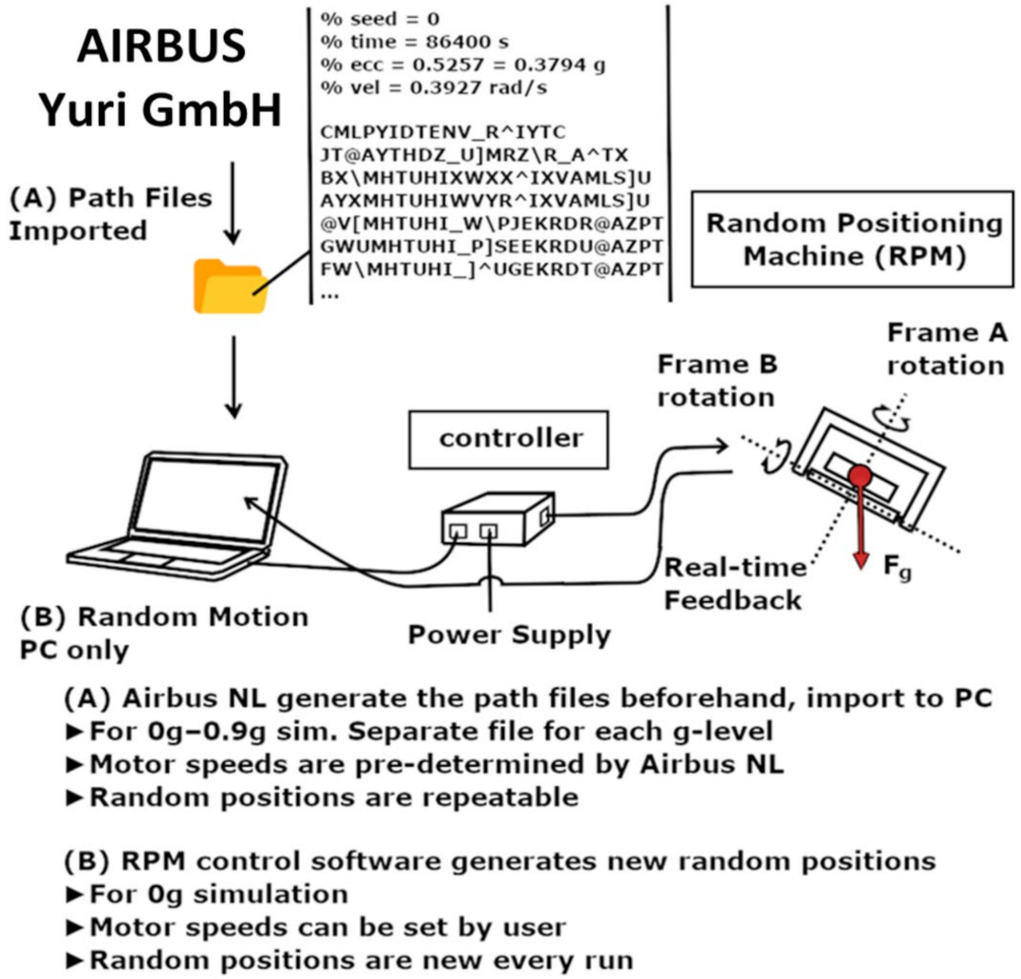

Supplementary Figure 3. The two motion modes are available for the RPM. Motion (A) requires the input of pre-determined path-files, available from Airbus NL/Yuri GmbH (https://www.yurigravity.com). These can be viewed or edited using Notepad++ source code editor (freeware, developer Don Ho (https://notepad-plus-plus.org/author)). Motion (B) independently and randomly runs, resulting in a net-zero simulation with additional options for control over rotation speed not available when using path files.

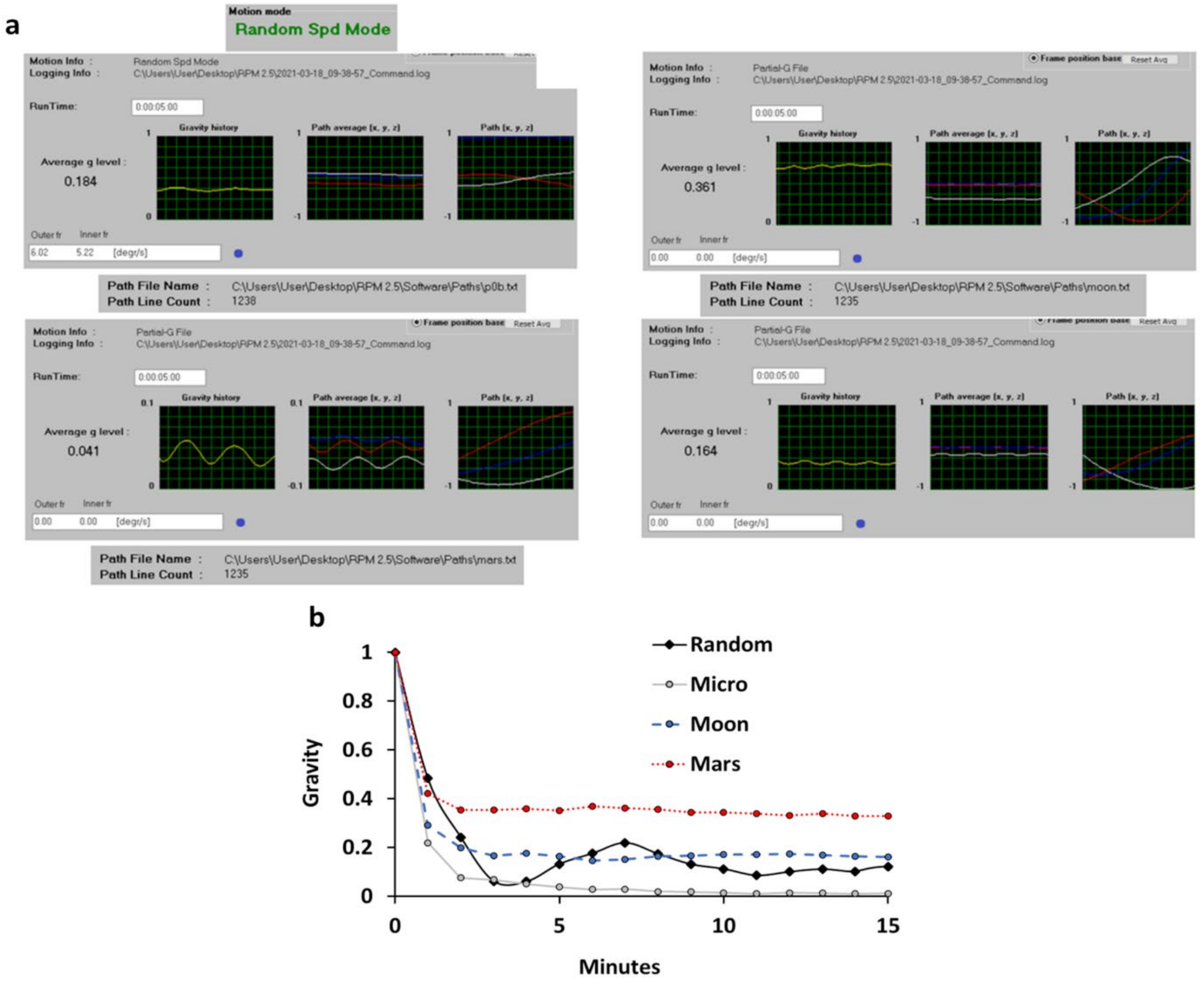

Supplementary Figure 4. (a) The software calculated vector-averaged simulated gravity (Fg) after 5 minutes for Random-motion mode and three path files: Zero Gravity, Mars Gravity, Moon Gravity. Also visible: frame paths, average path, and final vector average over time (gravity history). (b) Time-course plot of averaged simulated gravity level through the initial fifteen minutes beginning simulation in the RPM^{SW} using conventional random-motion (Random) to yield 0G and the following path-files: p0b.txt (Micro) (microgravity), mars.txt (Mars), moon.txt (Moon). While for Micro, Moon, and Mars the path files reached the desired gravity levels and stabilized in 3 to 5 minutes, it took more than an hour for Random Mode to reach 0.006G. These are original observations from our experiments, loaded with four T12 cell culture flasks.
